# Ureteral versus appendiceal Mitrofanoff channels: a retrospective analysis of functional outcomes and complications

**DOI:** 10.1186/s12893-026-03492-0

**Published:** 2026-02-09

**Authors:** Mohamed Mansy, Mostafa Kotb, Mohamed Abokandil, Amr Salama, Mohamed Gamal, Saber Waheeb, Mostafa Zain

**Affiliations:** 1 Nile of Hope Hospital for Congenital Anomalies, Alexandria, Egypt; 2 Port Said University, Port Said, Egypt; 3 Alexandria University, Alexandria, Egypt

**Keywords:** Appendicovesicostomy, Mitrofanoff channel, Clean intermittent catheterization

## Abstract

**Introduction:**

The Mitrofanoff principal entails creating a continent catheterizable channel, traditionally the appendix. In patients with nonfunctioning kidney, the ureter of this kidney can be an alternative This study aims to compare outcomes of ureter versus appendiceal Mitrofanoff channels (MCs).

**Patients and methods:**

We retrospectively reviewed patients who underwent MC creation between January 2020 and June 2024 for patients with neurogenic bladder, exstrophy–epispadias complex, prune belly syndrome, or posterior urethral valves. Group A included patients who had a ureter as MC (*n* = 11), while Group B included those with an appendiceal MC (*n* = 33). Data on demographics, operative details, complications, and continence outcomes were collected and analyzed.

**Results:**

Forty-four patients (Male patients = 24) were included, with a median age of 13.0 years (IQR 9.0–15.0) in Group A and 10.0 years (IQR 7.0–12.0) in Group B (*p* = 0.002). Myelomeningocele was the most common diagnosis in both groups. Bladder augmentation was performed in 81.8% of patients in each group, predominantly with ileo-cystoplasty. Satisfactory clean intermittent catheterization (CIC) without complications was achieved in 54.4% of Group A and 57.5% of Group B. Stomal stenosis occurred more frequently in Group B (18.2% vs. 9.1%, *p* = 0.003), while meatal granuloma was more common in Group A (27.3% vs. 9.1%, *p* = 0.043). Other complications included urine leakage, painful catheterization, catheterization difficulty, and intestinal obstruction, with no significant intergroup differences.

**Conclusion:**

Ureter as an MC offers a valid option for continent urinary diversion, demonstrating comparable continence outcomes as compared to appendiceal MC. The ureter represents a feasible and reliable alternative conduit in patients with a nonfunctioning kidney.

## Introduction

The concept of clean intermittent catheterization (CIC), introduced by Lapides in 1972, represented a paradigm shift in managing neurogenic bladder and urinary incontinence [1]. However, long-term urethral catheterization remains associated with complications such as blockage, leakage, urethral trauma or stricture, and bacterial colonization [2]. In 1980, Mitrofanoff proposed the appendicovesicostomy, offering an alternative route for bladder drainage and revolutionizing care for patients unable to catheterize via the urethra due to strictures, severe postural deformities, or conditions like spina bifida and scoliosis [3].

Since its inception, the Mitrofanoff (MF) principle of continent cutaneous urinary diversion has evolved, retaining its core concept: creating a catheterizable conduit to a low-pressure bladder reservoir via an accessible stoma [4]. While the appendix is most commonly used, other tissues as the ureter, the ileum, the sigmoid colon, and even the vas deferens or the fallopian tube, have been employed, each with distinct advantages and limitations [5].

A notable challenge of using a refluxing ureter as a Mitrofanoff channel (MC) lies in its need for division at two points, one for the stoma and another at the ureterovesical junction for reimplantation, which risks compromising its blood supply and predisposes to stenosis or segmental loss [6]. Some have proposed omitting ureteral reimplantation in patients undergoing simultaneous bladder augmentation, as the increased capacity and low intravesical pressure mitigate reflux risk [5, 7] [Zhang 2016]. This study examines the outcomes of utilizing a refluxing ureter without reimplantation as an MC, compared with the conventional appendiceal MC.

### Patients and methods

A retrospective study was conducted to evaluate the outcome of using the ureter as an MC in comparison to using the appendix. The study included patients who required an MC in our hospital during the period from January 2020 to June 2024 due to neurogenic bladder, exstrophy epispadias complex, prune belly syndrome, and posterior urethral valve.

In patients with the previously mentioned conditions, bladder augmentation with a concomitant MC was indicated in cases with (1) detrusor leak point pressure (DLPP) of 40 cm H_2_O or higher with evidence of upper tract damage, (2) intractable incontinence secondary to detrusor overactivity, (3) abnormal compliance associated with intractable urinary incontinence or upper tract deterioration or (4) recurrent urinary tract infection (UTI) due to vesicoureteral reflux (VUR). In patients with a neurogenic acontractile huge bladder with adequate capacity, an MC was performed alone without bladder augmentation. The absolute contraindication for MC was the inability to perform regular intermittent catheterization by the patient or the caregivers from the natural urethral orifice. All patients were operated on by the same surgical team in our institute throughout the study period.

The study was approved by the Ethics Committee of the Faculty of Medicine, Alexandria University. As a retrospective review of medical records, no additional interventions were performed, and patient care was not influenced by the study. All data were anonymized to maintain patient confidentiality, and the study adhered to the principles of the Declaration of Helsinki.

Patients were divided into two groups according to the conduit used. Group A included patients with bilaterally functioning kidneys, in whom the appendix was used as the MC. On the other hand, Group B included patients with a unilateral nonfunctioning kidney, in whom the ipsilateral ureter was used as the MC.

### Preoperative evaluation

Preoperative evaluation included (1) review of the previous surgical history, (2) physical and mental capacity to perform intermittent catheterization (3) routine laboratory investigations, (4) Ultrasound (US) to detect hydronephrosis, ureteric dilatation, and gross bladder anatomy, (5) Voiding cystourethrogram (VCUG) to evaluate the presence of vesicoureteral reflux (VUR) and to assess bladder capacity and postvoiding residual volume, (6) DMSA scan to detect renal scarring (7) Urodynamic study in the form of pressure-flow study to assess bladder capacity, compliance, contractility, emptying, and coordination. Magnetic resonance urography (MRU) was performed in cases with complex pathology or previous multiple surgeries.

### Operative technique

In Group A, a lower midline incision was used. The caecum and ileum were mobilized, and the appendix with its mesentery was detached from the caecum. Appendiceal patency was confirmed using a 12-Fr feeding tube. The distal appendix was implanted into the bladder through a 2-cm submucosal tunnel to create a flap-valve mechanism, and the proximal end was exteriorized as a stoma at the umbilicus or right McBurney point according to appendiceal length.

In Group B, laparoscopic transperitoneal nephrectomy of the nonfunctioning kidney was performed. The ureter was mobilized to its vesical insertion with preservation of periureteric vascularity, and its length and caliber were assessed to ensure tension-free reach to the chosen stomal site. A subcutaneous tunnel was created, and the proximal ureter was matured as a catheterizable cutaneous stoma with a wide, well-vascularized, tension-free opening; a catheter was left in situ to maintain patency.

### Postoperative care

Postoperative management was reviewed retrospectively from medical records. In both groups, patients routinely received prophylactic antibiotics and were monitored for stoma viability, urinary leakage, and signs of infection. The catheter left in the stoma intraoperatively was typically maintained for 2–3 weeks to allow epithelialization and prevent stenosis, as per institutional practice. Before discharge, patients and caregivers were instructed on CIC technique. Follow-up data, when available, included outpatient visits assessing renal function, upper urinary tract imaging, stoma patency, and continence status.

### Data collection and analysis

Demographic, clinical, and operative data were extracted from medical records. Outcomes evaluated included operative time, hospital stay, early and late complications (e.g., stenosis, leakage, UTI), and need for revision. Data were tabulated and analyzed using descriptive statistics. Continuous variables were expressed as mean ± standard deviation, and categorical variables as frequencies and percentages. Comparative analysis between groups was performed using the appropriate statistical tests, with a p-value < 0.05 considered statistically significant.

## Results

In our study, 44 patients underwent MC procedure. Appendix was used in 35 patients (group A), while ureter was used in the remaining 11 patients (group B) (Table 1). Seven male patients underwent ureteric MC compared to seventeen male patients in the appendicovesicostomy (APV) group (63.6% vs. 51.5%, respectively). The median ± (IQR) age group for patients in group A was 13 years (9.0–15.0) compared to median (IQR) age of 10 years (7.0–12.0) in group B (*p* = 0.002).

**Table 1:** Comparisonbetween the two studied groups according to different parameters

	Appendix (*n* = 33)	Ureter (*n* = 11)	*P*
Sex			
Male	17 (51.5%)	7 (63.6%)	0.484
Female	16 (48.5%)	4 (36.4%)	
Age at operation (years)			
Min. – Max.	9.0–15.0	7.0–12.0	
Median (IQR)	13.0 (11.0–14.0)	10.0 (8.0–11.0)	
Primary pathology			
MMC	20 (60.6%)	9 (81.8%)	0.529
Bladder Exstrophy	6 (18.2%)	0 (0.0%)	
PUV	3 (9.1%)	1 (9.1%)	
Hinnman’s syndrome	2 (6.1%)	0 (0.0%)	
Cloaca & ARM	1 (3.0%)	0 (0.0%)	
Prune belly syndrome	1 (3.0%)	1 (9.1%)	
Operative time (min)**			
Min. – Max.	75.0–103.0	68.0–80	< 0.001^*^
Median (IQR)	88 (77.5–95.0)	78 (70.0–80.0)	
Bladder augmentation			
No augmentation	6 (18.2%)	2 (18.2)	^FE^p= 1.000
Bladder augmentation	27 (81.8%)	9 (81.8%)	
Part of bowel used in augmentation			
No Augmentation	6 (18.2%)	2 (18.2%)	1.000
Ileum	25 (75.8%)	9 (81.8%)	
Ileocecal	2 (6.1%)	0 (0.0%)	
Sigmoid			
Ureter used			
Normal	33 (100.0%)	0 (0.0%)	< 0.001^*^
Refluxing	0 (0.0%)	11 (100.0%)	
Not assessed	0 (0.0%)	0 (0.0%)	

*IQR*, Inter quartile range *SD, *Standard deviation *t,* Student t-test χ^2^, Chi square test *FET*, Fisher Exact test

**Time taken in fashioning the appendix and the ureter as MC, not including the rest of the operative steps

*p*: *p* value for comparing between the two studied groups*: Statistically significant at *p* ≤ 0.05

Pathology subtypes in both groups included myelomeningiocele (20 in group A and 9 in group B), bladder exstrophy in 6 patients (group A only), posterior urethral valve (3 in group A and 1 in group B), Hinnman’s syndrome in only 2 patients from group A, cloaca in one case from group A and 1 patient in each group diagnosed with prune belly syndrome. Bladder augmentation was done in 27 patients (81.8%) from group A and 9 patients (81.8%) from group B. Ileocystoplasty was done in all augmented patients in group B and majority of patients from group A (9,100.0% vs. 25,75.8%, respectively), while the ileocecum was used in 2 patients only from group A.

**Fig. 1:**
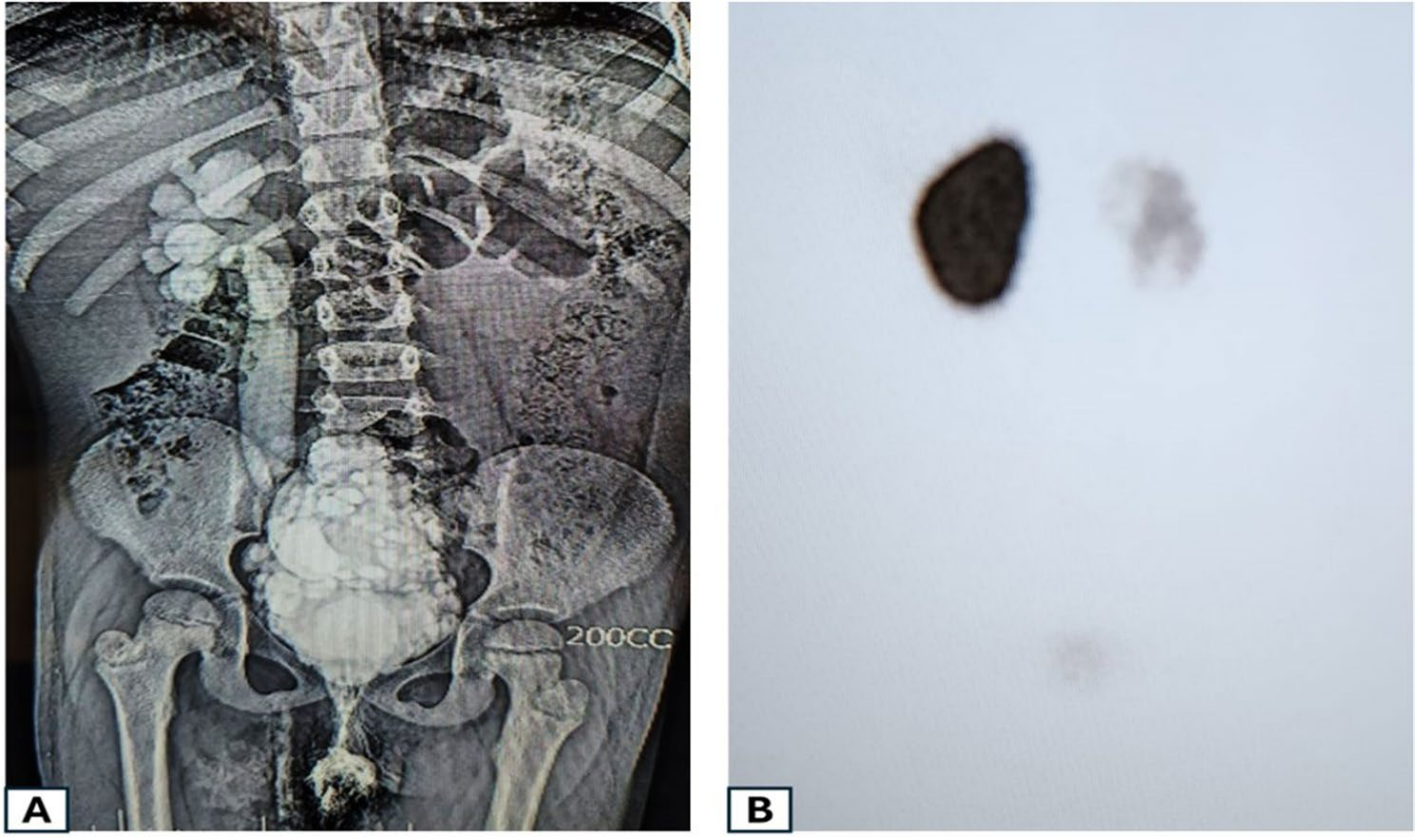
An example from the first group: An 8-year-old girl with a neurogenic bladder following MMC repair. **A **VCUG demonstrated a “Christmas tree” bladder with grade 5 VUR on the right side. **B **This right kidney was found to be non-functioning by DMSA renogram

**Fig. 2:**
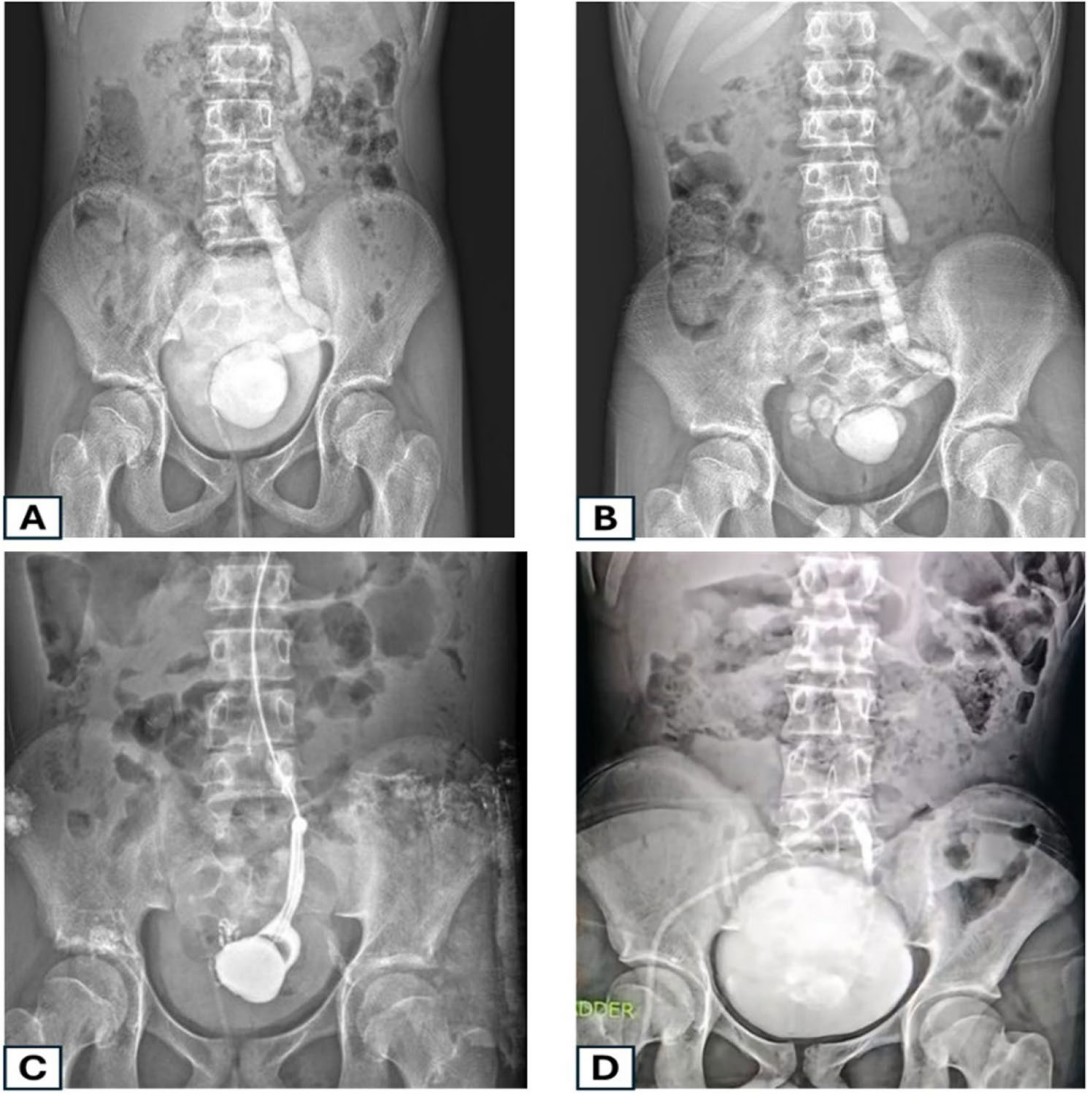
An example from the first group: An 11-year-old boy with a-contractile bladder following MMC repair. **A **VCUG demonstrated a large ureterocele with grade 3 reflux on the left side associated with a non-functioning kidney. **B **non-evacuation of the ureterocele and ureter after voiding. **C **Intraoperative descending study through the ureter showing filling of the ureterocele with no passage of the contrast into the bladder. **D **Postoperative descending contrast study of a ureteric Mitrofanoff after excision of the ureterocele. In our study, associated Malone was used in 9 patients in group A compared to 21 in group B (81.0%) vs. (63.6%) respectively. (*p* = 0.456)

In group B, the ureter used as MC was a refluxing ureter in all included patients (*n* = 11). In this group, the right ureter was used in eight patients (72.7%) (Fig. 1). In these eight patients, the right iliac region was used as a site for the MF channel. The remaining three patients had the left ureter used, and the stoma was positioned in the left lower quadrant region (Fig. 2). Around two-third of the patients in group A had the stoma in the right iliac region, the remaining patients in this group had it positioned in the umbilicus (33.3%). Operative time was significantly shorter in Group B than in Group A (Mann–Whitney U = 332.5, *p* < 0.001).

The mean follow-up duration was 21.1 ± 8.40 months in group A and 19.18 ± 8.80 months in group B. Satisfactory CIC was achieved in 19 children in the first group and 6 children from the second group (54.4% vs. 57.5%) with no reported complications.

**Fig. 3:**
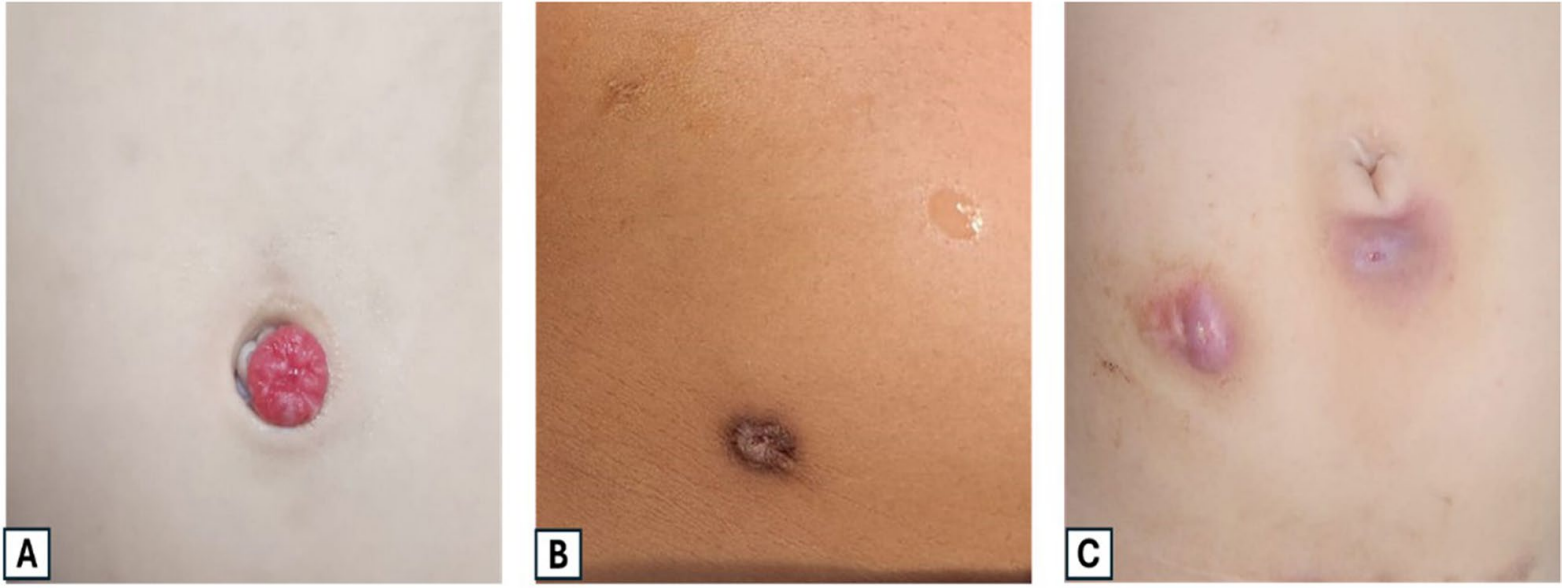
Examples of complications associated with the external opening of the Mitrofanoff stoma. **A **Granuloma formation at the stoma site (raised, red, fungating tissue). **B **Stenosis of the stoma; narrowed and contracted external opening following appendicovesicostomy. **C **Perimeatal inflammation causing painful catheterization

Postoperative complications included stenosis, leakage, meatal granuloma formation, painful catheterization, directional difficulty and intestinal obstruction (Table 2). In our statistical analysis, we found that stenosis was a significant incident in group A compared to group B (6, 18.2% vs. 1, 9.1%; *p* = 0.003) and was managed by meatal refashioning. Meatal granuloma occurred equally in both groups (3 patients each, 9.1% and 27.3% in group A and B, respectively) and this was found to be also statistically significant (*p* = 0.043) (Fig. 3). Excision of the granuloma and maturation of the meatus was sufficient to manage these cases.

**Table 2:** Comparison between the two studied groups according to operative considerations and complications

	Appendix (*n* = 33)	Ureter (*n* = 11)	*P*
Contralateral side			
Normal	33 (100.0%)	11 (100.0%)	–
Refluxing	0 (0.0%)	0 (0.0%)	
Not assessed	0 (0.0%)	0 (0.0%)	
Side			
Right	33 (100.0%)	8 (72.7%)	< 0.001^*^
Left	0 (0.0%)	3 (27.3%)	
Site of Mitrofanoff			
Umbilical	11 (33.3%)	0 (0.0%)	^FE^p= 0.041^*^
Right Iliac	22 (66.7%)	8 (72.7%)	
Left Iliac	0 (0.0%)	3 (27.3%)	
Associated Malone			
No	12 (36.4%)	2 (18.2%)	^FE^p= 0.456
Yes	21 (63.6%)	9 (81.8%)	
Complications			
Stenosis	6 (18.2%)	1 (9.1%)	*p* = 0.003* *p* = 0.63 *p* = 0.043 *p* = 0.63 *p* = 0.87
Leakage	2 (6.1%)	0 (0.0%)	
Meatal Granuloma	3 (9.1%)	3 (27.3%)	
Painful catheterization	2 (6.1%)	0 (0.0%)	
Directional Difficulty	0 (0.0%)	1 (9.1%)	
Intestinal obstruction	1 (3.03%)	0 (0.0)	
No complication	19 (57.5%)	6 (54.5%)	
Secondary intervention			
No	23 (69.7%)	6 (54.5%)	^FE^p= 0.287
Yes	10 (30.3%)	5 (45.5%)	
Follow-up duration			
Min. – Max.	6.0–36.0	8.0–36.0	0.529
Mean ± SD.	21.1 ± 8.40	19.18 ± 8.80	
Median (IQR)	22.0 (15.0–28.0)	17.0 (13.0–25.0)	

*IQR*, Inter quartile range *SD,* Standard deviation *t,* Student t-test χ^2^, Chi square test *FET*, Fisher Exact test

*p*: *p* value for comparing between the two studied groups

*: Statistically significant at *p* ≤ 0.05

**Fig. 4:**
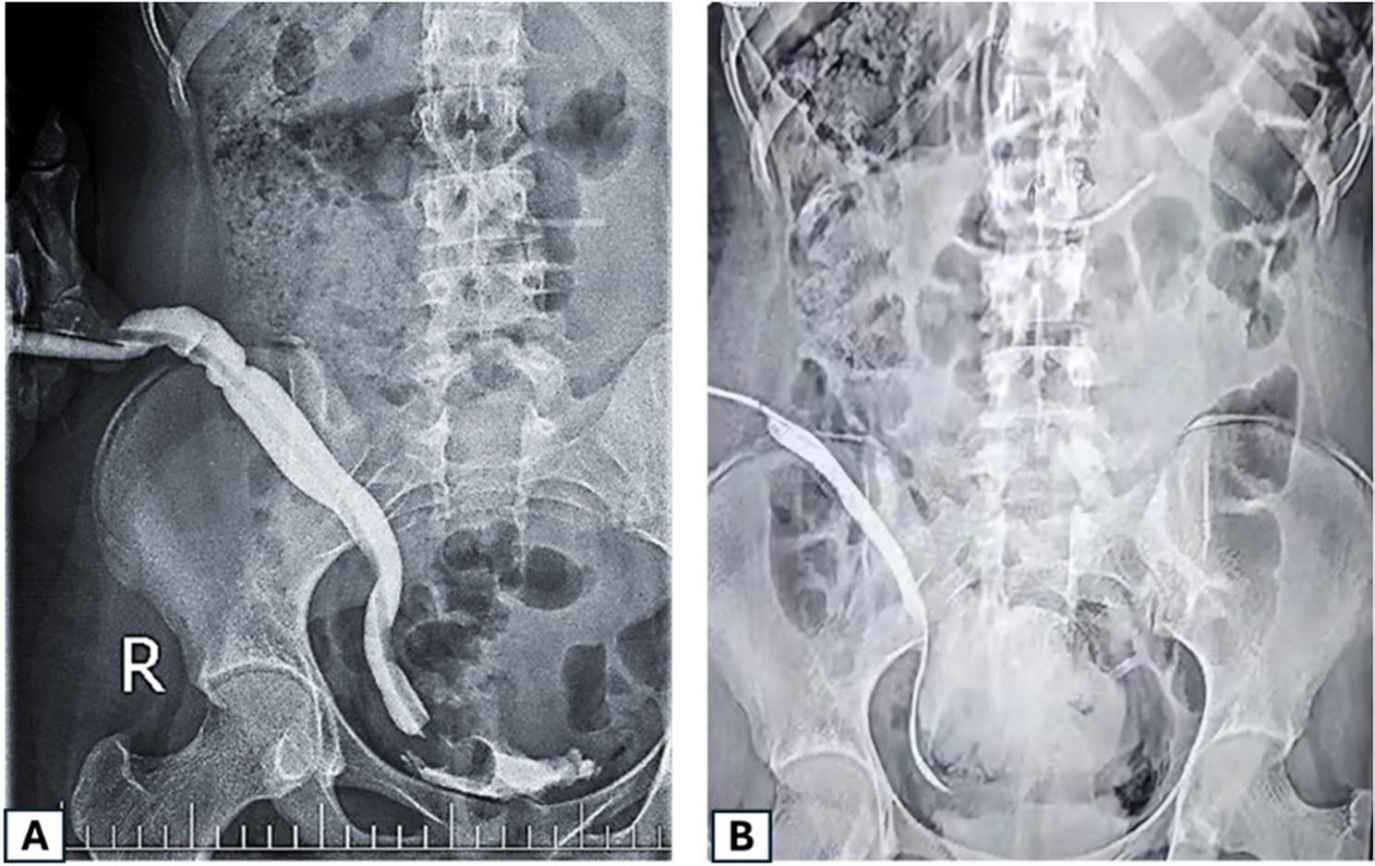
(**A**) A descending contrast study of a ureteric Mitrofanoff showing a dilated, redundant ureter that caused catheter insertion difficulties. Surgical correction involved straightening and excision of the excess ureteric length. **B **Postoperative descending contrast study

Urine leakage through the MF was found in 2 patients in group A (6.1%). This was managed successfully with anticholinergics and three-hour CIC. Directional difficulty during catheterization happened in one patient in group B due to a dilated tortuous ureter; secondary intervention was done in this patient to excise part of the tortuous ureter to correct this tortuosity (Fig. 4). Peri-meatal inflammation caused painful catheterization in 2 patients from group A (6.1%); however, it was managed by local application of analgesics and anti-inflammatory medications. Lastly, postoperative adhesive intestinal obstruction occurred in one patient from group A and was managed by adhesiolysis (3.03%).

## Discussion

The appendicular continent cystostomy was first described by Mitrofanoff in 1980, using the appendix as a conduit between the bladder and skin [2]. This allowed the bladder to be emptied by a route other than the urethra and was a further revolutionary step in the field of urinary incontinence management following the earlier introduction of clean intermittent self-catheterization by Lapides [3].

Since the original description by Mitrofanoff, several variations have been reported as the procedure evolved. The underlying principle of the MF procedure is the creation of a conduit going into a low-pressure reservoir, which can empty through clean intermittent catheterization through an easily accessible stoma [1, 2]. The indications for this procedure include refractory neurogenic bladder (with or without myelomeningocele), refractory idiopathic bladder dysfunction, severe urethral stricture disease or difficult self-catheterization as an adjunct to reconstruction in congenital urogenital abnormalities (cloacal exstrophy, posterior urethral valves, epispadias, and prune belly syndrome) [4, 8].

The appendix is commonly used as an MC because of its suitable length, flexibility, and ease of mobilization; however, its use is limited in some patients. Prior appendectomy, congenital absence, or previous reconstructive use as in Malone antegrade continence enema may preclude availability [9]. An inadequate length or caliber can prevent creation of a suitable stoma, especially in obese patients or when a long subcutaneous tunnel is required [8, 9]. Because the appendix has a single end-artery, excessive mobilization increases the risk of ischemia, necrosis, or stenosis. Stomal stenosis at the cutaneous or bladder anastomosis is relatively common and may require dilation or revision [10]. Persistent mucous production can interfere with catheterization and increase the risk of urinary tract infection [7, 9]. Additionally, anatomical variations may add operative complexity, and the use of the appendix eliminates its availability for future reconstructive procedures [10, 11].

In situations where the appendix is unavailable or unsuitable, several alternative catheterizable conduits have been described. The Monti procedure, introduced by Monti et al.., involves isolating and detubularizing a short segment of ileum, which is then tubularized to form a conduit of sufficient length and diameter for catheterization [12]. This technique provides a reliable option, particularly for patients requiring a longer channel. The Yang-Monti modification further enhances versatility by joining multiple short ileal segments end-to-end, facilitating the creation of a longer conduit without sacrificing excessive bowel length [13]. Both techniques have demonstrated favorable continence rates but are associated with potential complications such as stomal stenosis, mucus production, and bowel-related morbidity [14]. In selected cases, a dilated, nonfunctioning ureter can be repurposed as a catheterizable channel, offering a native, vascularized conduit [15]. However, ureteric conduits are limited by variable length and caliber and are generally reserved for cases involving a nonfunctional renal unit or duplicated collecting systems [16].

Operative time is an important consideration when selecting a conduit for MF catheterizable stoma creation in pediatric patients, especially in complex reconstructive cases. In our series, patients with documented nonfunctioning kidney undergoing a MF procedure +/- augmentation, the ureter of the non-functioning kidney was preferably used as an MC over the use of appendix and the operative time was generally shorter although not statistically significant. Likewise, Frank et al. reported shorter operative time in ureter MC cohort with no significant difference in the operative time between both groups [17]. This is attributed to using a pre-dilated, non-functioning ureter can streamline the procedure by avoiding bowel mobilization or appendiceal harvest, though additional time may be required if the ureter needs extensive dissection.

Earlier reports mentioned that ureter MC had more major complications, this included urine leak, the need of complete revision, or even higher rate of stenosis when compared to the appendix [18]. In other series, there was no statistically significant difference between the complication rates of both ureteral MC and APV [17]. However, our series showed that complication rates were much lower.

Stomal stenosis is among the most common long-term complications following creation of continent catheterizable channels in children, often necessitating repeated dilations or revision surgeries. Van Savage et al., found a stenosis rate of 13% in appendiceal channels, which is close to our results, making it the most common channel-related complication observed [16]. On the other side, ureteric MC appear to demonstrate lower rates of stomal stenosis in our study with only one case developed stenosis from the ureter MC cohort. Similarly, none of the 18 patients who underwent MC using ureter developed stenosis as demonstrated by Van Savage et al., suggesting that ureteric tissue may be somewhat less prone to stenosis than appendiceal conduits [16]. From our perspective, it is possibly attributed to the less dissection of the ureter owing to its longer length and easier mobilization to the abdominal wall compared to appendix; both factors reduce the risk of ischemia and tension and hence the incidence of stenosis.

Meatal (stomal) granuloma formation is a relatively common minor complication following MF catheterizable stomas, irrespective of the conduit type used. Reported incidence varies by conduit, patient factors, and follow-up duration. The incidence of meatal granulomas has been reported to be 8% and is most commonly encountered in appendiceal MF [19], which is very close to our results (9%). These patients usually present by difficulty in catheterization, bleeding during or after catheterization [19]. Postoperative adhesive intestinal obstruction occurred in one patient from group B and was managed by adhesiolysis. Gowda et al., reported in his study that included 65 patients having MF procedures; 8% returned to OR theatre in the peri-operative period for bleeding and bowel obstruction [20].

The conventional way of treating VUR is to improve the competence of uretero-vesical valve by ureteric reimplantation. In our study we used a refluxing ureter of nonfunctioning kidney without reimplantation as an MF in group A to avoid any affection for the ureteric vascularity. Tapre et al., recommended not to re-implant the refluxing ureter used as an MC to prevent vascular damage to the ureteric segment and to retain a virgin uretero-vesical junction [8].

In group A, 9 of our patients had radiological or cystometrographic features of high-pressure, small bladder in addition to unilateral VUR so it was important to increase bladder capacity and decrease the bladder pressure by bladder augmentation. The other 2 cases had large a-contractile bladder in addition to unilateral VUR so bladder augmentation was not indicated. Urine leakage did not appear in any patient in group A which is similar to the results of Tapre et al., who demonstrated that the refluxing ureter could be used as a MF without reimplantation in most children who required bladder augmentation as a part of their urinary reconstruction [8].

We found that dexterity can be a limitation to some patients, for example in the three patients with the left ureter used as MF in the left lower quadrant, right-handed patient had initial difficulties in doing CIC; however, with training and rehabilitation they were able to use his left hand in CIC.

## Limitations

This study is limited by a significant age difference between groups and a small sample size—particularly in the ureteric cohort—reducing statistical power and limiting comparative validity. As these cases are rare, the findings should be considered preliminary. Larger, prospective or multicenter studies with longer follow-up are needed to better define outcome differences.

## Conclusions

The use of the ureter of a non-functioning kidney can be a good option as an MF channel, with satisfactory results and acceptable complication rate. Using ureter is technically easier with less operative time. Its usage in selected scenarios can lessen the complication rate with guaranteed comparable outcomes.

## Data Availability

The datasets used and/or analyzed during the current study are available from the corresponding author upon reasonable request.

## Abbreviations

APV: Appendicovesicostomy
CIC: Clean intermittent catheterization
MC: Mitrofanoff channel
MF: Mitrofanoff
VUR: vesicoureteral reﬂux
